# Preparation of polyetherimide membrane from non-toxic solvents for the separation of hydrogen from methane

**DOI:** 10.1186/s13065-018-0449-7

**Published:** 2018-07-10

**Authors:** Yousef Alqaheem, Abdulaziz Alomair, Abdulwahab Alhendi, Sharifah Alkandari, Nusrat Tanoli, Nourah Alnajdi, Andrés Quesada-Peréz

**Affiliations:** Petroleum Research Center, Kuwait Institute for Scientific Research, Ahmadi, Kuwait

**Keywords:** Membrane, Polyetherimide, Nontoxic solvents, Hydrogen, Methane

## Abstract

Polymeric membranes are usually prepared from solvents like *n*-methylpyrrolidone (NMP) because of the strong dissolving power and high boiling point. Yet, the solvent is costly, toxic and has environmental issues. In this work, nontoxic solvents such as methyl l-lactate, ethyl lactate, propylene carbonate, tributyl *o*-acetylcitrate, tributyl citrate, triethyl phosphate, and γ-butyrolactone (GBL) were introduced during membrane preparation. It was found that all the solvents were unable to dissolve polyetherimide except GBL. The membranes made by GBL and NMP were evaluated for gas separation, and they have almost similar hydrogen-to-methane selectivity, but, hydrogen permeance was better in NMP membranes.

## Introduction

Polymeric membranes were introduced in the oil/gas industry in the 1980s for the separation of hydrogen from natural gas [1]. The technology was successful because of the low operating cost and zero emission [2]. Later, the applications were expanded to include carbon dioxide capture, air separation, and recovery of volatile organic compounds [3]. Today, the technology can compete with other gas-separation processes such as cryogenic distillation and pressure swing adsorption (PSA) [4].

The preparation method of polymeric membranes plays a critical role on the membrane performance, and solvent selection is one of the key variables [5]. For example, some authors reported a remarkable increase in the membrane permeability with different solvents, and this was related to the change in membrane morphology such as pore size and membrane thickness [6].

The most used technique for membrane preparation is by phase inversion [2, 7–10]. The method consists mainly of four steps: (1) dilution of the polymer in a solvent with a defined polymer-to-solvent ratio, (2) heating and mixing the solution to obtain a homogenous mixture, (3) tape casting the solution by an applicator with a preset thickness, and (4) immersing the solution in a water bath to form the polymer film. The membrane is then left to dry before operation.

Solvents like *n*-methylpyrrolidone (NMP) is commonly used for membrane preparation because of the strong dissolving power and high boiling point of 202 °C [2, 11]. Despite these features, the solvent has some drawbacks related to cost and toxicity. Working with NMP without personal protective equipment (PPE) may result in severe skin burns and serious eye injury [12]. Furthermore, the solvent may damage the reproductive system or the unborn child. NMP can also harm the aquatic life; and therefore, it requires special treatment before disposal.

On the other hand, nontoxic solvents are available, and some of them have similar properties to toxic ones. Some examples are methyl l-lactate (ML), ethyl lactate (EL), propylene carbonate (PC), tributyl *o*-acetylcitrate (ATBC), tributyl citrate (TBC), triethyl phosphate (TEP), and γ-butyrolactone (GBL). Some of these solvents were investigated for the preparation of porous membranes for liquid separation, and the results were promising. For example, cellulose acetate (CA) was prepared using NMP and ML for ultrafiltration and the developed membranes had similar performance in terms of molecular weight cutoff (MWCO) and rejection values [13]. In addition, polyvinylidene fluoride (PVDF) membranes were made for microfiltration (MF) using TEP, and the membranes have similar pore structure compared to NMP [14].

Table 1 shows physical properties and prices of the nontoxic solvents compared to NMP. In terms of density, EL has a density of 1.03 g/cm^3^ which is identical to NMP. On the other hand, GBL has a very close boiling point (204 °C) to NMP. All the solvents have a lower price compared to NMP; therefore, their usage will have a significant reduction in the production cost of the membrane.

**Table 1:** Physical properties and cost of non-toxic solvents [15, 16]

Solvent	Density (g/cm^3^)	Boiling point (°C)	Estimated price ($/ton)
*n*-Methylpyrrolidone (NMP)	1.03	202	2700
Methyl l-lactate (ML)	1.09	145	2250
Ethyl lactate (EL)	1.03	154	1200
Propylene carbonate (PC)	1.20	242	1400
Tributyl *o*-acetylcitrate (ATBC)	1.05	331	2050
Tributyl citrate (TBC)	1.04	170	1700
Triethyl phosphate (TEP)	1.07	215	2050
γ-Butyrolactone (γ-BL)	1.13	204	1600

To best of our knowledge, nontoxic solvents are rarely used for the preparation of gas separation membranes. It is not necessarily that the new solvents will work; because, for gas separation, a dense membrane is needed instead of a porous one. Having a defect-free membrane is not an easy task because any change in the solvent properties can greatly affect the membrane morphology. Moreover, the change in solvent selection may require a modification in the preparation procedure to determine the optimum polymer-to-solvent ratio.

In this paper, polyetherimide (PEI) membranes were prepared using different solvents such as NMP, ML, EL, PC, ATBC, TBC, TEP, and GBL. Hansen model was used to predict the dissolving power of those solvents. The membranes were evaluated for gas separation by measuring hydrogen and methane permeation. After the operation, the membranes were characterized by scanning electron microscopy (SEM), electron-dispersive X-ray spectroscopy (EDX) and X-ray diffraction (XRD) to observe any changes in the membrane structure.

## Hansen model and solvents selection

Hansen model was used to predict if the new solvents will be suitable for dissolving polyetherimide. The model is based on calculating the polymer–solvent distance (*d*) using solubility parameters as follows:

1 $$d = \sqrt {\left[ {\delta_{{d\left( {solvent} \right)}} - \delta_{{d\left( {polymer} \right)}} } \right]^{2} + \left[ {\delta_{{p\left( {solvent} \right)}} - \delta_{{p\left( {polymer} \right)}} } \right]^{2} + \left[ {\delta_{{h\left( {solvent} \right)}} - \delta_{{h\left( {polymer} \right)}} } \right]^{2} }$$

where *δ*_d_, *δ*_p_, and *δ*_h_ are the solubility parameters of dispersion component, dipolar intermolecular component and hydrogen bond component, respectively. The lower the value of polymer-solvent distance, the more power the solvent will have to dissolve the polymer [17].

Table 2 shows the calculated polymer-solvent distance for PEI with various solvents. The data give an indication that ATBC, ML, and TBC will dissolve PEI better than NMP. It should be noted that Hansen model is not always correct; because, polymer morphology, solvent molecular size, and temperature are not taken into consideration [18]. Therefore, experimental work is needed to confirm that the solvent is suitable for PEI.

**Table 2:** Hansen solubility parameters for various solvents and the calculated PEI-solvent distance [17]

Compound	*δ*_*h*_ (MPa)^1/2^	*δ*_*d*_ (MPa)^1/2^	*δ*_*p*_ (MPa)^1/2^	*δ*_*(solvent*–*PEI)*_ (MPa)^1/2^
Polyetherimide (PEI)	6.4	17.7	6.0	–
*n*-Methylpyrrolidone (NMP)	7.2	18.4	12.3	6.39
Methyl l-lactate (ML)	10.2	15.8	6.5	4.28
Ethyl lactate (EL)	12.5	16.0	7.6	6.53
Propylene carbonate (PC)	4.1	20.0	18.0	12.43
Tributyl *o*-acetylcitrate (ATBC)	6.2	15.4	4.1	2.99
Tributyl citrate (TBC)	10.1	16.6	3.8	4.44
Triethyl phosphate (TEP)	9.2	16.8	11.5	6.24
γ-Butyrolactone (γ-BL)	7.4	19.0	16.6	10.73

## Membrane preparation

The membranes were prepared by phase inversion method. PEI was dissolved in the solvent with different concentrations of 23, 27, and 30 wt%. The solution was mixed and heated at 60 °C for 24 h. If PEI did not dissolve, the temperature was increased gradually to 140 °C. After that, the solution was tape casted on a glass sheet using an applicator to form a membrane with a thickness of 300 μm. The glass was then immersed in a water bath for 24 h to participate the polymer and remove the solvent. The membrane was removed from the bath and kept to dry for 24 h. This procedure is widely used by many researchers [19].

Table 3 shows that up to 140 °C, only GBL was capable of fully dissolving PEI. Actually, PEI started to dissolve in GBL at a temperature of 100 °C. ATBC however, managed to dissolve only few amounts of PEI at 140 °C. Increasing the temperature to 160 °C did not help in increasing the solubility; instead, the solution turned black due to decomposition of the polymer.

**Table 3:** Effect of temperature on the solubility of polyetherimide in different solvents

Solvent	Was PEI fully soluble in the solvent?				
	60 °C	80 °C	100 °C	120 °C	140 °C
*n*-Methylpyrrolidone (NMP)	Y		Y	Y	Y
Methyl l-lactate (ML)	N	N	N	N	N
Ethyl lactate (EL)	N	N	N	N	N
Propylene carbonate (PC)	N	N	N	N	N
Tributyl *o*-acetylcitrate (ATBC)	N	N	N	N	N
Tributyl citrate (TBC)	N	N	N	N	N
Triethlyl phosphate (TEP)	N	N	N	N	N
γ-Butyrolactone (GBL)	N	N	Y	Y	Y

Other solvents like ML, EL, PC, TBC, and TEP did not dissolve any PEI. This result conflicts with the conclusion from Hansen model; nevertheless, this was expected because the model ignored the polymer morphology and solvent molecular size, and these parameters greatly affect the solubility.

From this point, PEI membranes were prepared using GBL and NMP. For GBL, membranes with PEI concentrations of 23 and 27 wt% were successfully prepared but for 30 wt% PEI, it was difficult to tape cast the solution because the polymer immediately participated due to temperature-induced phase (TIP) separation. PEI concentration in the solution was calculated based on weight:

2 $$PEI\; ( {\text{wt)}}\% = \frac{{W_{PEI} }}{{W_{Solvent} + W_{PEI} }}$$

where $W_{PEI}$ and $W_{Solvent}$, are the weights of PEI and solvent, respectively.

## Membrane evaluation

Four different membranes were tested for hydrogen and methane permeation. Two membranes were prepared by NMP with PEI concentration of 23 wt% (NMP-23) and 27 wt% (NMP-27); while the other two were prepared by GBL with PEI concentration of 23 wt% (GBL-23) and 27 wt% (GBL-27). The operating conditions were set to 25 °C with a feed flow rate of 100 L h^−1^. Feed pressure was varied from 3 to 10 bar. Permeance (*P*) was calculated by the following equation:

3 $$P\varvec{ } ( {\text{GPU)}} = \frac{{V_{p} }}{{A\varvec{ }\Delta P}}$$

where *V*_*p*_ is the permeate volume flowrate, *A* is the active membrane area of 12.6 cm^2^, and $\Delta P$ is the pressure difference between the feed and permeate sides. *V*_*p*_ was measured using a membrane gas-permeation cell (Convergence Inspector Neptunus). Hydrogen permeance ($P_{{H_{2} }}$) and methane permeance ($P_{{CH_{4} }}$) were used to calculate the selectivity (*α*):

4 $$\upalpha_{{{\rm H}_{2} /{\rm CH}_{4} }} = \frac{{P_{{{\rm H}_{2} }} }}{{P_{{{\rm CH}_{4} }} }}$$

Permeation and selectivity data of the prepared membranes are given in Table 4. In terms of permeance, NMP resulted in membranes with higher permeance compared to GBL. For example, at a concentration of 23 wt% PEI and a feed pressure of 10 bar, NMP membrane gave a hydrogen permeance of 580 GPU; while GBL membrane gave a permeance of 153 GPU. In terms of selectivity, overall, GBL membranes had a slightly better selectivity compared to NMP. The maximum selectivity was 3.3 achieved at 3 bar with PEI concentration of 27 wt%. On the other hand, NMP membrane resulted in a selectivity of 3.0; but hydrogen permeance again was very high compared to what GBL membrane achieved.

**Table 4:** Hydrogen and methane permeation data for polyetherimide membrane made from NMP and GBL at 25 °C and different feed pressures

Sample	PEI wt%	Feed pressure	Permeance (GPU)		Selectivity
			H_2_	CH_4_	
NMP-23	23	3	464.2	248.9	1.9
		5	493.3	280.8	1.8
		7	530.2	311.9	1.7
		10	580.0	355.6	1.6
NMP-27	27	3	43.7	14.5	3.0
		5	47.1	16.1	2.9
		7	48.7	17.3	2.8
		10	51.1	19.8	2.6
GBL-23	23	3	109.3	43.2	2.5
		5	129.3	56.9	2.3
		7	141.5	80.8	1.8
		10	153.5	94.3	1.6
GBL-27	27	3	1.1	0.3	3.3
		5	1.8	0.7	2.6
		7	1.9	0.8	2.4
		10	2.0	1.0	2.0

## Membrane characterization

**Fig. 1:**
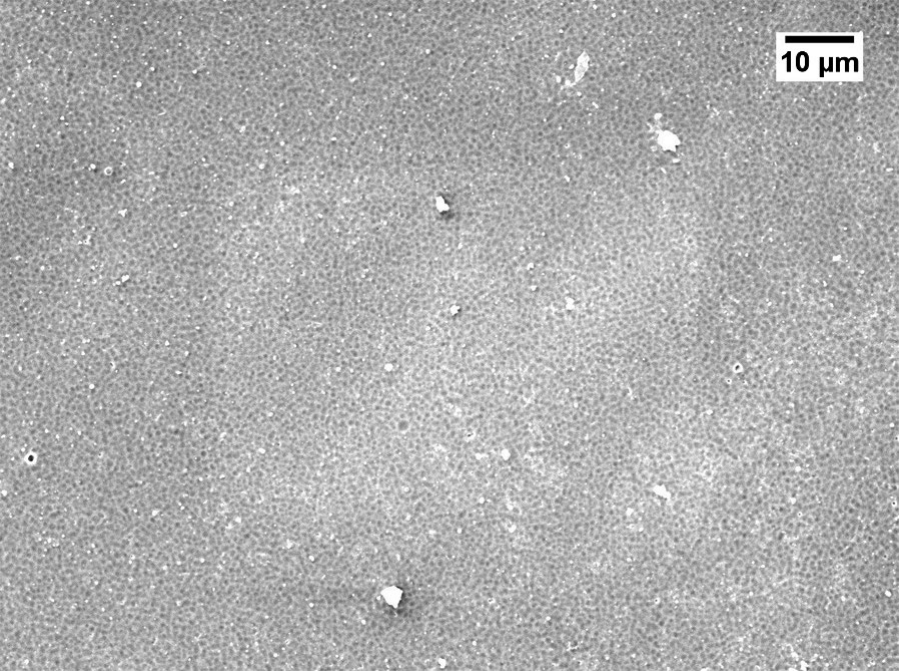
SEM image of PEI membrane made by NMP and 23 wt% PEI

**Fig. 2:**
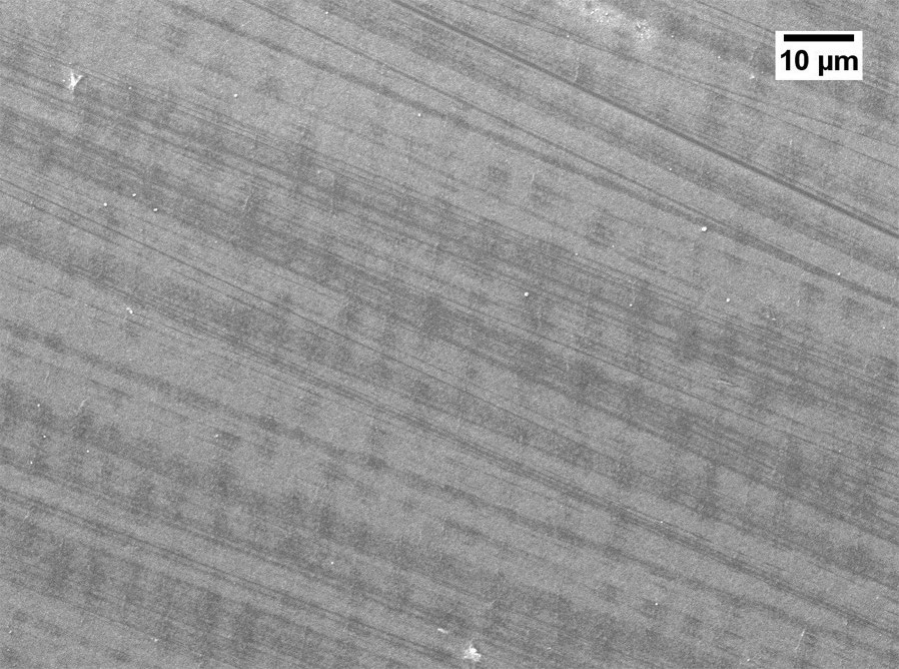
SEM image of PEI membrane made by NMP and 27 wt% PEI

**Fig. 3:**
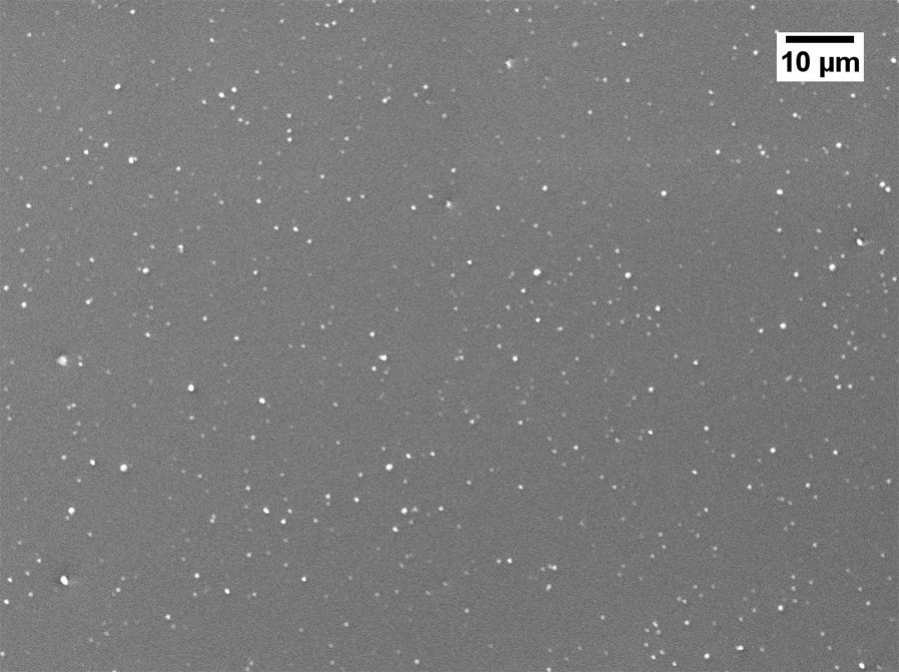
SEM image of PEI membrane made by GBL and 23 wt% PEI

**Fig. 4:**
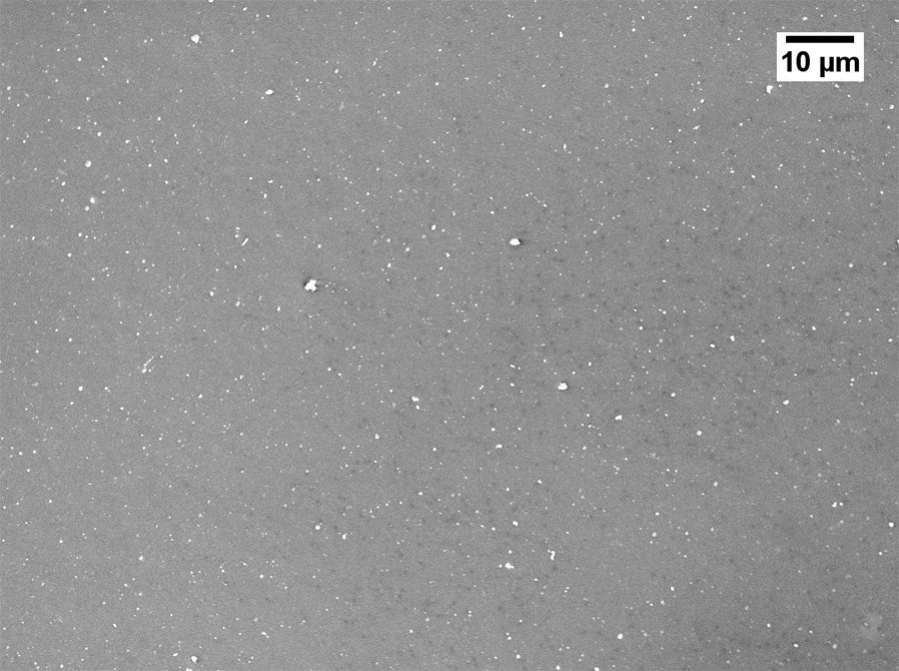
SEM image of PEI membrane made by GBL and 27 wt% PEI

**Fig. 5:**
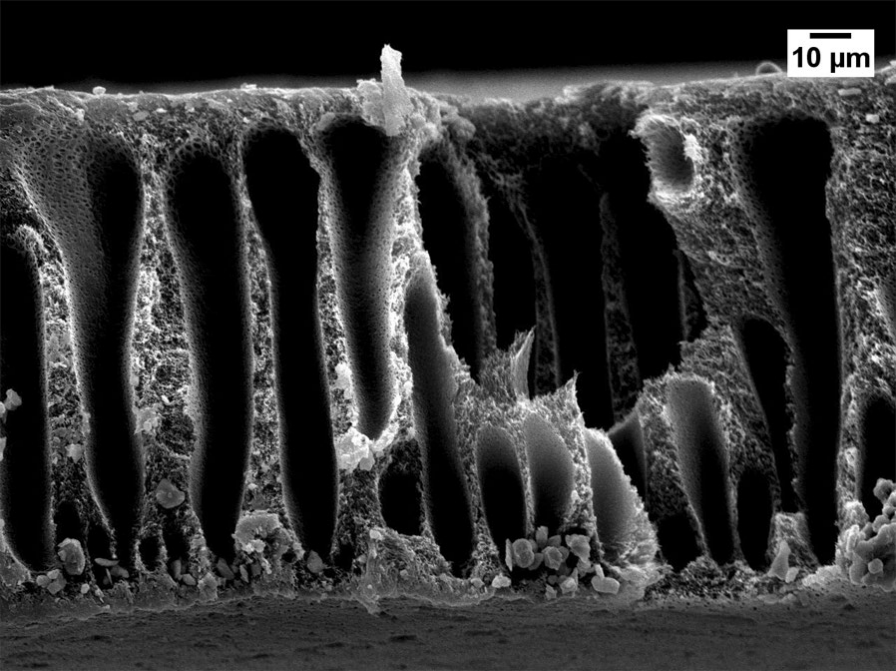
Cross-section image of NMP membrane made by 23 wt% PEI

**Fig. 6:**
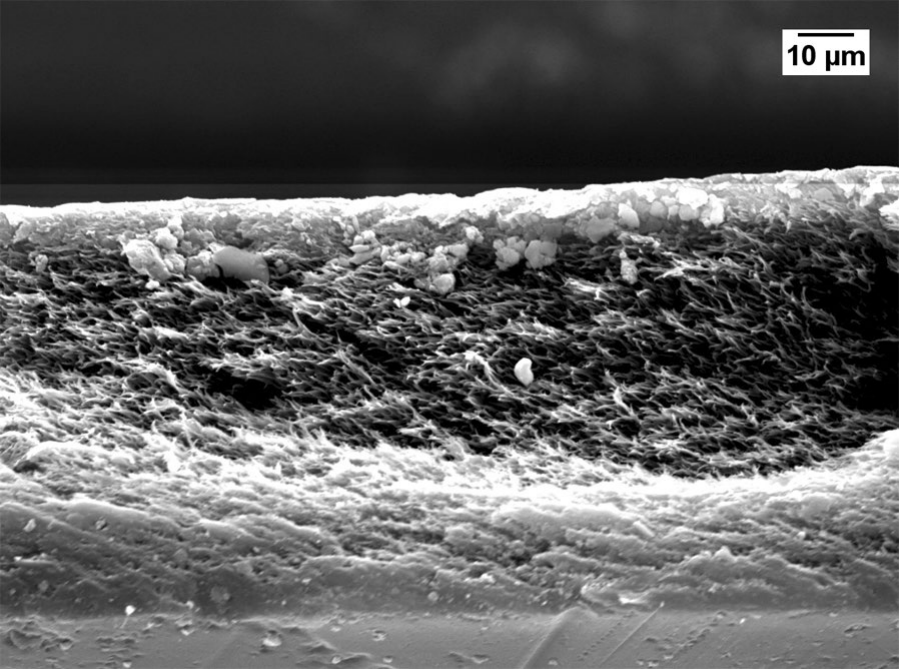
Cross-section image of NMP membrane made by 27 wt% PEI

**Fig. 7:**
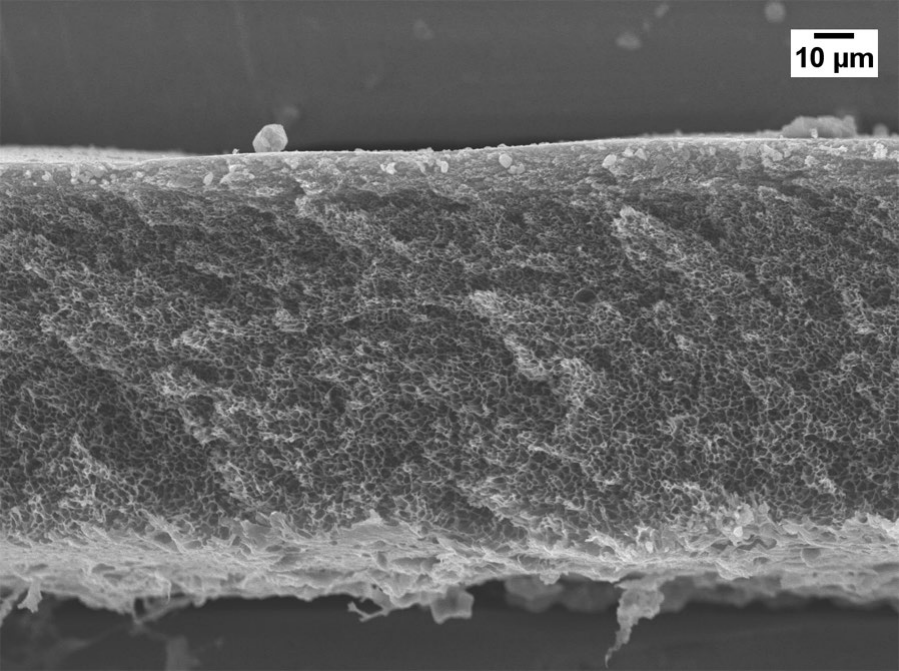
Cross-section image of GBL membrane made by 23 wt% PEI

**Fig. 8:**
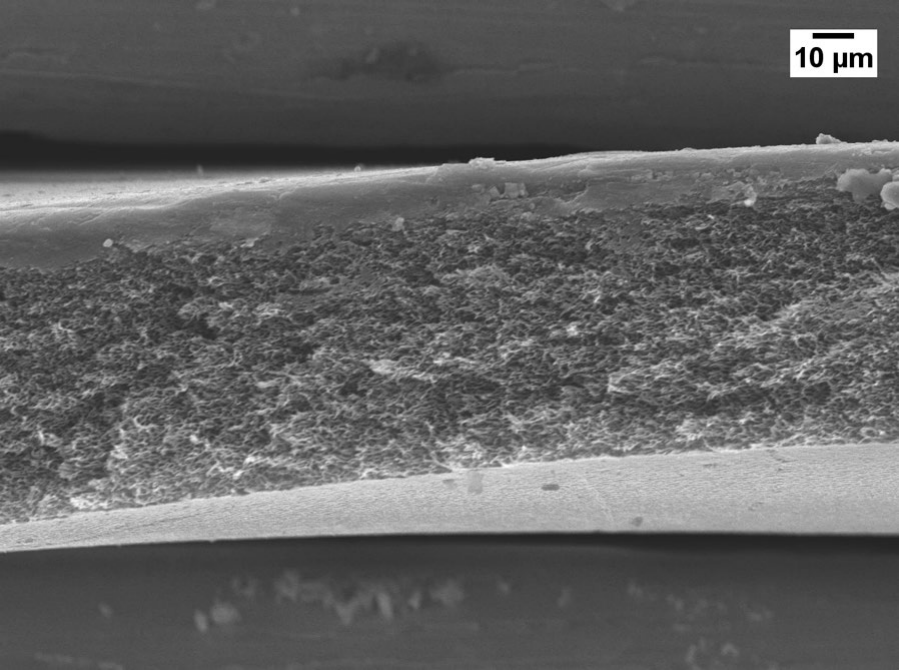
Cross-section image of GBL membrane made by 27 wt% PEI

Severe reduction of the membrane permeability due to the use of GBL was also noticed by other researchers [20, 21]. To investigate why GBL membranes have low permeance, SEM (JEOL, JSM-IT300) was used to examine the membrane surface that was exposed to the gases. The samples were cut using liquid nitrogen and Figs. 1, 2, 3, 4 show that all the membranes have a dense structure with no defects. Another factor the can control the permeability is the membrane thickness. During tape casting, the applicator was set to form a membrane with a thickness of 300 μm, but the produced membranes should have a lower thickness because of the solvent exchange and only 23 to 27 wt% of PEI was used. Table 5 shows the thickness of the developed membranes by NMP and GBL. Membranes produced by NMP had an average thickness of 140 μm; while GBL resulted in membranes with a thickness of 80 μm. It is worth mentioning that the whole membrane structure is not always dense because of the evolution of solvent that causes a formation of both porous and dense layers [10]. The dense layer acts mainly as the selective barrier but the porous layer can also affect the gas mobility. SEM was used to examine the cross-section surface of the membranes and it was found that NMP membranes have a thickness of 6.5 and 8.7 μm for PEI concentration of 23 and 27 wt%, respectively. The increase in thickness of the dense layer with the increase in PEI concentration was also confirmed by others [22]. As given in Figs. 5 and 6, the porous structure of NMP membranes has large voids and this can be linked to the fast evolution of NMP solvent during the solvent exchange. A similar structure having these voids was also reported in other studies [10]. On the other hand, for GBL membranes, the porous structure has a lower porosity indicating a low precipitation time during the solvent exchange. Because of this slow participation rate, GBL membranes have a thicker, more dense layer of 9.6 and 12.5 μm for PEI concentration of 23 and 27 wt%, respectively (Figs. 7, 8). This densified layer could be related to the poor interaction between GBL and PEI which prevented chain stretching and caused coiling [23]. Based on SEM, the low permeance in GBL membranes can be related to the large thickness of the dense layer. Furthermore, the low porosity of the porous structure could also slowdown the gas movement. Because polymeric membranes have the tradeoff between permeability and selectivity, the low permeance resulted in improvement in the selectivity as methane molecules took longer to pass through the membrane [24].

**Table 5:** Thickness of the final membranes after tape casting thickness of 300 μm

Sample	PEI wt%	Total thickness (μm)	Dense layer thickness (μm)
NMP-23	23	150	6.5
NMP-27	27	130	8.7
GBL-23	23	85	9.6
GBL-27	27	75	12.5

EDX (Oxford Instrumentation, INCA X-ACT) was used to determine the chemical composition of the membranes. The chemical formula of PEI is (C_37_H_24_O_6_N_2_)_*n*_, so it is expected to detect carbon, hydrogen, oxygen, and nitrogen. However, due to the limitation of EDX setup, only carbon and oxygen were detected. Data is given in Table 6 and all the membranes have a nearly identical composition of carbon (85 wt%) and oxygen (15 wt%). This confirms that there were no impurities introduced during membrane preparation.

**Table 6:** EDX data for PEI membranes prepared from NMP and GBL

Sample	PEI wt%	Carbon wt%	Oxygen wt%
NMP-23	23	84.8	15.2
NMP-27	27	84.9	15.1
GBL-23	23	84.9	15.1
GBL-27	27	84.6	15.4

**Fig. 9:**
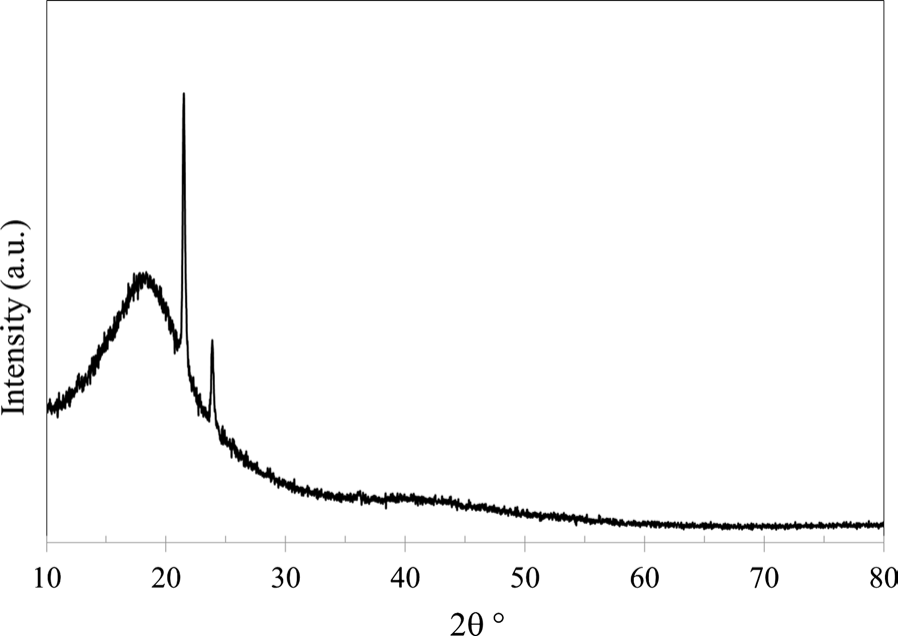
XRD analysis of NMP membrane made by 23 wt% PEI

**Fig. 10:**
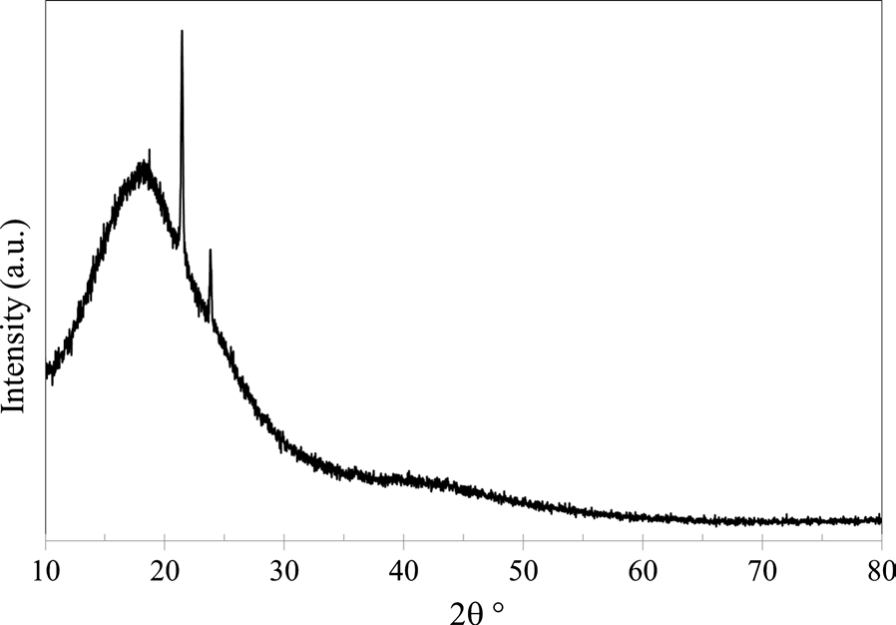
XRD analysis of NMP membrane made by 27 wt% PEI

**Fig. 11:**
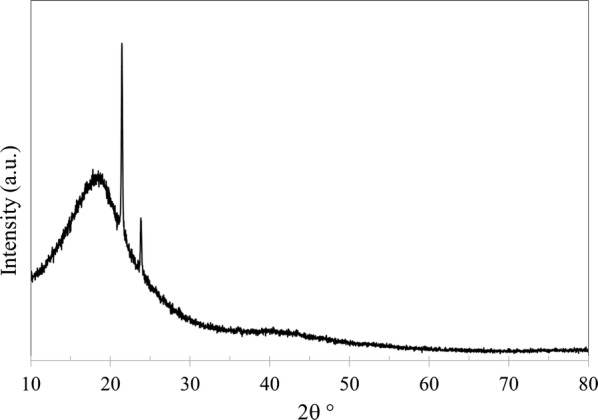
XRD analysis of GBL membrane made by 23 wt% PEI

**Fig. 12:**
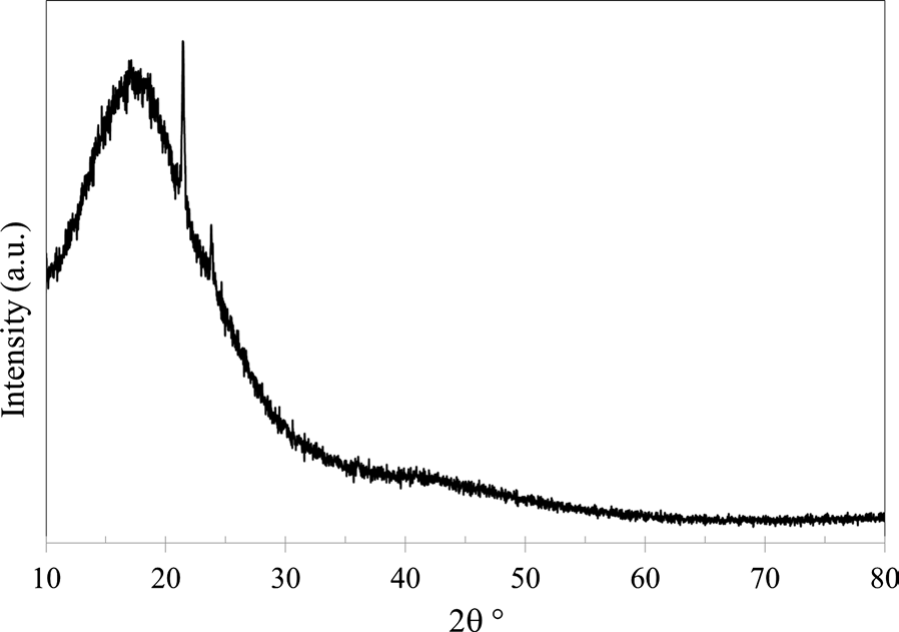
XRD analysis of GBL membrane made by 27 wt% PEI

In addition to SEM and EDX, the membranes were analyzed using XRD (PANalytical, Empyrean XE) to observe any changes in the structure crystallinity. Furthermore, XRD was used to calculate d-space (*d*) which represents the distance between polymer chains. Bragg’s law was applied to determine d-space using:

5 $$n = 2d\text{sin}\theta$$

where *n* is the order of reflection, *λ* is the wavelength of the diffractometer and *θ* is XRD angle of the maximum peak. Figures 9, 10, 11 and 12 shows XRD data of NMP and GBL membranes and NMP membranes have slightly higher intensity particularly for PEI concentration of 27 wt% meaning that the structure is more crystallized. d-Space values of NMP and GBL membranes are presented in Table 7 and similar values were obtained indicating that GBL did not alter the chain distance.

**Table 7:** d-Space values of NMP and GBL and membranes based on XRD data

Sample	PEI wt%	d-Space (Å)
NMP-23	23	4.15
NMP-27	27	4.14
GBL-23	23	4.14
GBL-27	27	4.15

It should be noted that there are many parameters in the preparation method that may improve the permeability of GBL membranes such as evaporation duration, coagulation media, and coagulation bath temperature. Evaporation duration is the time after tape casting in which the film is transported to the coagulation bath. It was found by Mohamad et al. that reducing this duration improved the permeability of PEI membrane for carbon dioxide separation [10]. Furthermore, water, methanol, ethanol, and isopropanol are usually selected as the bath media but Mohamad et al. study showed that water performs better compared to other media. However, the experiments were conducted using NMP as a solvent, not GBL. Use of alcohols may reduce the participation time of PEI and this may reduce the thickness of the dense layer of GBL membranes for better permeability [22]. Bath temperature has also a great influence on the membrane structure and it was found that high bath temperature increases the diffusion of solvent and non-solvent due to the rapid molecules movement and this caused formation of a porous structure with a lower thickness of the dense layer [22, 25].

## Conclusion

NMP is one of the traditional solvents for polymeric membrane preparation. The chemical has a strong solvent power with a high boiling point making it an excellent solvent for many polymers. However, the solvent is toxic and has many health and environmental issues. In this work, nontoxic solvents such as ML, PC, ATBC, TBC, TEP, and GBL were investigated for the preparation of PEI membrane for gas separation. Hansen model showed that some of the new solvents will have a very good solubility for PEI but practically, only GBL was capable of dissolving PEI. This was explained by the limitation of Hansen model due to polymer morphology and solvent molecular size. Membranes with PEI concentration of 23 and 27 wt% were prepared by NMP and GBL. These membranes were evaluated for hydrogen and methane permeation, and data showed that membranes made by GBL had slightly better hydrogen-to-methane selectivity compared to NMP membranes. However, the permeance was significantly reduced when GBL was used as a solvent. SEM revealed that GBL membranes have a more densified layer that limited the gas transport. Also, the poor solubility of GBL may resulted in a lower interaction between polymer and solvent causing a slow precipitation rate during the solvent exchange. The low permeability of GBL membranes  may be improved by optimizing other factors in the preparation method such as evaporation duration, coagulation bath media and bath temperature. Increasing the participation duration, using alcohol as a bath media and increasing the bath temperature may reduce the thickness of the dense layer for higher permeability.
